# A HER2-specific Modified Fc Fragment (Fcab) Induces Antitumor Effects Through Degradation of HER2 and Apoptosis

**DOI:** 10.1038/mt.2015.127

**Published:** 2015-08-25

**Authors:** Kin-Mei Leung, Sarah Batey, Robert Rowlands, Samine J Isaac, Phil Jones, Victoria Drewett, Joana Carvalho, Miguel Gaspar, Sarah Weller, Melanie Medcalf, Mateusz M Wydro, Robert Pegram, Geert C Mudde, Anton Bauer, Kevin Moulder, Max Woisetschläger, Mihriban Tuna, John S Haurum, Haijun Sun

**Affiliations:** 1 F-star Biotechnology Ltd, Babraham Research Campus, Cambridge, UK

## Abstract

FS102 is a HER2-specific Fcab (Fc fragment with antigen binding), which binds HER2 with high affinity and recognizes an epitope that does not overlap with those of trastuzumab or pertuzumab. In tumor cells that express high levels of HER2, FS102 caused profound HER2 internalization and degradation leading to tumor cell apoptosis. The antitumor effect of FS102 in patient-derived xenografts (PDXs) correlated strongly with the HER2 amplification status of the tumors. Superior activity of FS102 over trastuzumab or the combination of trastuzumab and pertuzumab was observed *in vitro* and *in vivo* when the gene copy number of HER2 was equal to or exceeded 10 per cell based on quantitative polymerase chain reaction (qPCR). Thus, FS102 induced complete and sustained tumor regression in a significant proportion of HER2-high PDX tumor models. We hypothesize that the unique structure and/or epitope of FS102 enables the Fcab to internalize and degrade cell surface HER2 more efficiently than standard of care antibodies. In turn, increased depletion of HER2 commits the cells to apoptosis as a result of oncogene shock. FS102 has the potential of a biomarker-driven therapeutic that derives superior antitumor effects from a unique mechanism-of-action in tumor cells which are oncogenically addicted to the HER2 pathway due to overexpression.

## Introduction

HER2 is an oncoprotein in the ERBB receptor family. Activation of this receptor family induces potent signaling through the mitogen-activated protein kinase (MAPK) and phosphatidylinositol 3-kinase (PI3K) pathways which promote tumor cell growth and survival.^1^ HER2 does not have a known ligand and exists in an “open” configuration with its dimerization interface accessible in the native state.^2,3^ This property makes HER2 the preferred dimerization partner of the other ERBB receptors.^4^ Heterodimers containing HER2 are poorly internalized and degraded, a phenomenon that is more prominent when HER2 is overexpressed.^5^

HER2 is overexpressed in 20–30% of breast and gastric cancers and expression is correlated with a poor prognosis.^6,7,8,9^ A number of HER2-targeted agents have been developed, including trastuzumab, pertuzumab, trastuzumab emtansine (T-DM1), and lapatinib. However, a large unmet medical need remains due to intrinsic or acquired resistance. For example, the response rate to T-DM1 in HER2-positive advanced breast cancer patients was only less than 50%. Even for those who responded, the median progression-free survival was less than a year.^10^

**Figure 1:**
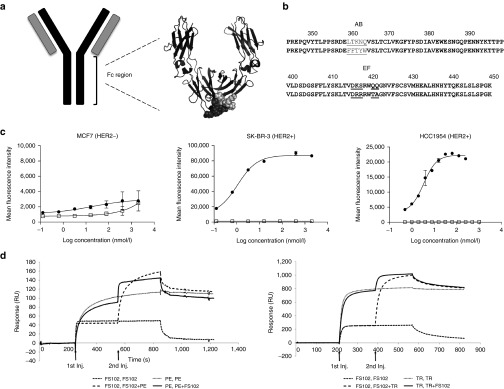
**Fcab structure and binding characterization of FS102**. (**a**) Graphic representation of an IgG with the Fc region magnified to show the crystal structure of the WT Fcab. The light and dark gray space fills in the crystal structure denote the AB and EF loops respectively. (**b**) Amino acid sequence comparison of FS102 (bottom) to WT Fcab (top). Differences are underlined. (**c**) Specific binding of FS102 to cell surface HER2 demonstrated by flow cytometry. Solid circles: FS102; Open Squares: WT Fcab. Each data point is the average of duplicate samples (error bars: standard deviation). (**d**) Competition in binding to HER2 ECD among FS102, trastuzumab (TR), and pertuzumab (PE) determined using BIAcore. Left: Competition to HER2-bound PE, right: Competition to HER2-bound TR. The legend denotes the two analytes that were injected sequentially. The two injections are indicated by arrows. Injection of FS102 results in the same response unit (RU) gain on a clean HER2 coated surface (---) as a PE or TR saturated surface (—). Similarly, TR or PE give the same response unit change when injected on a clean HER2 surface (___) or a FS102 saturated surface (---). Together these data show that FS102 does not compete for binding to HER2 with TR or PE. WT, wild type.

An Fc fragment with antigen binding (Fcab) is a 50 kDa homodimeric antibody fragment format derived from the constant domain of human IgG1 (residues 238–478 by Kabat numbering). The engineering of the Fcab has been described previously by replacing amino acid sequences in the human IgG1 Fc fragment located at the C-terminal structural loops in the CH3 domain to generate antigen binding sites (**Figure 1a**).^11^ The Fcab scaffold typically retains effector functions as well as the long half-life comparable to wild-type (WT) Fcab (the unmodified corresponding sequence of the constant region of human IgG1) at one third of the size of an IgG. Such properties set the Fcab apart from other antibody scaffolds of similar or smaller size.

Here we report the discovery and preclinical activity of a novel biologic, FS102, a HER2-targeting Fcab. FS102 has a unique mechanism-of-action that involves induction of tumor cell apoptosis, and exhibits superior antitumor activity compared to standard of care antibodies in xenograft tumor models preselected according to a novel biomarker strategy. The results presented suggest that FS102 has the potential to address unmet medical needs including those arising from resistance to trastuzumab-based therapies.

## Results

### Selection and production of an anti-HER2 Fcab

Experimental and computational stability studies were performed to assess the effects of randomization of distinct regions within the CH3 domain of human IgG1 Fc fragment.^12^ The identified permissive residues (358–362 and 413–419 Kabat numbering; referred to as AB and EF loops) were randomized to construct a yeast *Saccharomyces cerevisiae* surface display library of Fc fragments.^13^ The amino acid substitutions were carried out by PCR using NNB-oligonucleotides.^11^ The corresponding isotype control of Fcabs is the unmodified constant region of human IgG1 denoted as wild-type (WT) Fcab. The library was screened for binding to HER2 extracellular domain (ECD), and the structural integrity of the positive clones confirmed by their uncompromised binding to anti-CH2, CD64, and Protein A (data not shown). The resulting FS102 contains a total of 9 amino acid substitutions in the AB and EF loop of the CH3 domain compared to WT Fcab (**Figure 1b**). Production of soluble FS102 was achieved by mammalian expression in transfected CHO cells followed by protein A purification. Compared to the WT Fcab, FS102 exhibited a similar symmetrical single-peak size exclusion chromatography profile, comparable binding affinity to Fc receptors, and the same first thermal transition *T*_*m*_1 (70.8 °C; **Supplementary Figure S1** and **Supplementary Table S1**). The mouse serum half-lives *t*_1/2_ of FS102 and the WT Fcab were approximately 60 and 40 hours, respectively. Taken together, these results indicate that the overall structural integrity of FS102 was not significantly altered by the induced mutations.

### FS102 is specific for HER2 and binds to a different epitope than trastuzumab or pertuzumab

FS102 bound to recombinant HER2 ECD-Fc fusion with an apparent *K*_D_ of 0.8 ± 0.3 nmol/l. This binding was specific to HER2. The *K*_D_ of trastuzumab and pertuzumab binding to HER2 was 0.4 ± 0.03 and 6.3 ± 0.9 nmol/l, respectively (**Supplementary Table S2**). The BIAcore specificity experiment showed that FS102 did not have any detectable binding to the other HER family receptor EGFR, HER3, HER4 (**Supplementary Figure S2**). FS102 binds to HER2 overexpressing cancer cell lines SK-BR-3 (EC50 = 1.1 nmol/l) and HCC1954 (EC50 = 3.3 nmol/l). There is no significant binding to MCF7, which does not overexpress HER2 (**Figure 1c**). In a HER2 binding competition experiment, FS102 did not compete with trastuzumab or pertuzumab, indicating that the epitope of FS102 does not overlap with those of trastuzumab and pertuzumab (**Figure 1d**). Despite having nonoverlapping epitopes, FS102 and trastuzumab exhibited no significant difference in the number of binding sites recognized on the surface of HCC1954 or the SK-BR-3 cells (**Supplementary Figure S2**).

### FS102 induces profound receptor internalization and degradation in cell lines expressing a high level of HER2

**Figure 2:**
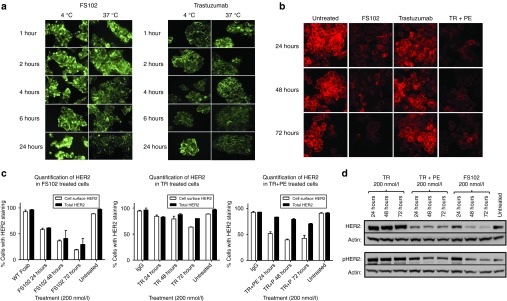
**Effects of antibodies on HER2 protein level and signaling**. TR, trastuzumab; PE, pertuzumab; TR+PE, 1:1 combination of trastuzumab and pertuzumab. (**a**) A time course of labeled FS102 and TR shows both can be internalized in BT474 cells. At 4 °C, both FS102 and TR show characteristic cell surface staining. At 37 °C, staining becomes increasingly more pixelated over time and loses the contour of the cells, suggesting internalization. (**b**) A time course showing membrane HER2 staining of SK-BR-3 cells after being treated with FS102, TR, and TR+PE (200 nmol/l, 37 °C) highlights the efficient removal of membrane HER2 by FS102 or TR+PE. (**c**) Quantification of HER2 staining confirms that FS102 is more effective in eliminating both membrane and total HER2 in SK-BR-3 cells than TR and TR+PE. All treatments were at 200 nmol/l and 37 °C. Membrane HER2 and total HER2 labeling were performed using unpermeabilized and permeabilized cells, respectively. Percentage of HER2-positive cells was calculated (error bars: standard deviations). (**d**) Western blot of SK-BR-3 cell lysates confirms significant reduction of total HER2 and pHER2 by FS102 and TR+PE. WT, wild type.

The effects of FS102 on HER2 protein level and HER2-mediated signaling as a function of time were investigated. In one study, labeled antibodies and Fcabs were incubated with HER2 expressing BT474 cells and the intensity of the labels was determined at various time points (**Figure 2a**). The results indicated that FS102 and trastuzumab were internalized at comparable rate and extent. In another study, unlabeled antibodies or Fcabs were incubated with SK-BR-3 cells, and membrane or total HER2 proteins were subsequently stained (**Figure 2b**,**c**). The results showed time-dependent depletion of membrane HER2 mediated by FS102, trastuzumab, and the combination of trastuzumab and pertuzumab. Maximal depletion was recorded after 72 hours of incubation. FS102, trastuzumab, and the combination of trastuzumab and pertuzumab reduced membrane HER2 levels by 81 ± 1.4, 35 ± 0.7, and 57.2 ± 6.0%, respectively. Meanwhile, significant time-dependent depletion of total HER2 was observed in cells treated with FS102 and the combination only. After 72 hours of incubation, FS102, trastuzumab, and the combination of trastuzumab and pertuzumab reduced total HER2 by 70 ± 16, 20 ± 3, and 29.1 ± 1.0%, respectively. To corroborate these findings, western blot of HER2 and pHER2 was conducted using the lysates of SK-BR-3 cells that had been treated with antibodies or Fcabs for 24, 48, or 72 hours. The results confirmed that total HER2 protein was reduced by both FS102 and to a lesser degree by the trastuzumab and pertuzumab combination but not by trastuzumab on its own (**Figure 2d**). For all treatment modalities, reduction in total HER2 closely correlate to that of the pHER2 (**Figure 2d**), suggesting a possible cause-effect relationship. The same time-course study was conducted using another HER2 overexpressing cell line HCC1954, and similar observations were made (data not shown).

### Apoptosis is the unique biological effect induced by FS102 and a prominent mechanism of action for tumor cell growth inhibition: *in vitro* assessment

**Figure 3:**
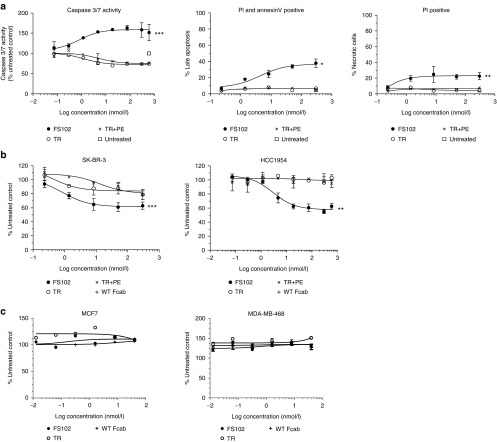
**FS102 induces apoptosis and reduces cell growth *in vitro***. SK-BR-3 cells were treated with FS102, trastuzumab (TR), or the trastuzumab and pertuzumab combination (TR+PE) for 5 days before analysis. Each data point is the average of duplicated samples (error bar: standard deviation). (**a**) FS102 induces apoptosis in HER2 overexpressing cell line, SK-BR-3. Shown by relative changes in caspase 3/7 activity, relative changes in the population of late apoptotic cells measured by Annexin V/propidium iodide (PI) staining, and relative changes in the population of necrotic cells measured by PI staining. (**b**) FS102 inhibits proliferation in HER2 overexpressing cell lines, as shown by relative decrease in SK-BR-3 and HCC1954 cell number after 5 days of treatment. (**c**) FS102 does not inhibit proliferation in HER2-negative cell lines as shown by relative changes in MCF7 and MDA-MB-468 cell number after 5 days of treatment. Statistical analysis performed using repeated one-way analysis of variance, * denoted *P* < 0.05; **P < 0.01; ***P < 0.001. WT, wild type.

Unlike trastuzumab, FS102 did not induce G1 cell cycle arrest or inhibit the shedding of HER2, (results not shown). Instead, FS102 treatment of SK-BR-3 cells activated caspase 3/7 in a dose-dependent manner with an EC_50_ of 0.8 nmol/l, whereas neither trastuzumab nor the combination of trastuzumab and pertuzumab increased caspase activity (**Figure 3a**). This indicates that induction of apoptosis is a unique mechanism of action of FS102. The apoptotic effect was apparent after relatively prolonged treatment (120 hours), corresponding to the hypothesis that the apoptosis observed is a consequence of the internalization and degradation of HER2.

A hallmark of apoptotic cells is compromised membrane integrity. Membrane flipping increases the exposure of extracellular phosphatidylserine. Permeabilized cell membrane allows propidium iodide to bind to nucleic acid. Staining of FS102-treated cells after 120 hours incubation showed the presence of extracellular phosphatidylserine and nucleic acid-bound propidium iodide in 38 ± 7 and 23 ± 5% of the cells, respectively (**Figure 3a**). Neither trastuzumab nor the combination of trastuzumab and pertuzumab produced any significant effects in the same experiments.

The pronounced apoptotic activity of FS102 correlated with a potent growth inhibition in a proliferation assay performed on cell lines with high HER2 gene amplification status. FS102 reduced SK-BR-3 cell numbers by 39 ± 8% after 5 days of incubation, compared to 22 ± 10 and 22 ± 3% by trastuzumab and the trastuzumab and pertuzumab combination, respectively (**Figure 3b**). This difference in potency was even more pronounced in HCC1954 cells: FS102 reduced the cell number by 47 ± 5% while the other two agents were ineffective (**Figure 3b**). To confirm that these activities of FS102 are target-specific, the same study was repeated using tumor cell lines in which HER2 is not amplified (MCF7 and MDA-MB-468). As expected, FS102 did not show any significant antiproliferative activity in these two cell lines (**Figure 3c**).

### FS102 induces complete tumor regression and tumor cell apoptosis: *in vivo* assessment

**Figure 4:**
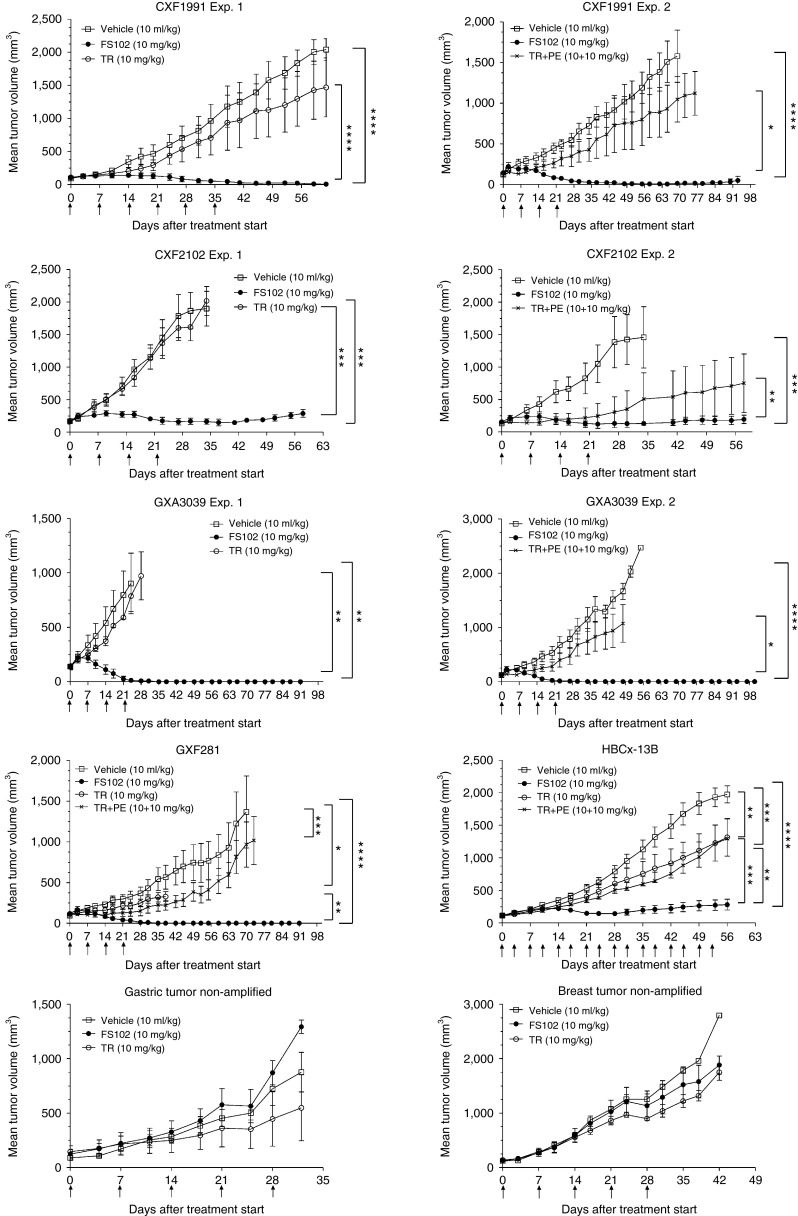
**Tumor growth in patient-derived xenograft (PDX) models in response to HER2 treatments**. Graphs showing mean tumor volumes of PDX treated with FS102, trastuzumab (TR), or the trastuzumab and pertuzumab combination (TR+PE) (error bar: standard deviation). PDX with HER2 gene amplification: CXF1991 and CXF2102: colorectal tumors; GXA3039 and GXF281: gastric tumors; HBCx-13B: breast tumor. PDX without HER2 amplification: gastric tumor nonamplified, breast tumor nonamplified. Treatment administration schedule is shown by arrows. Statistical analysis performed using repeated one-way analysis of variance, * denoted *P* < 0.05; ** denoted *P* < 0.01; *** denoted *P* < 0.001; **** denoted *P* < 0.0001.

The antitumor activity of FS102 was tested *in vivo* using mice implanted with HER2-positive PDX tumors. Remarkably, in one model of colorectal cancer (CXF1991) and two models of gastric cancer (GXA3039 and GXF281), FS102 induced complete tumor regression after 4–5 weekly treatments at 10 mg/kg and no tumor regrowth was observed during a 13-week follow-up period. In contrast, trastuzumab or the trastuzumab and pertuzumab combination did not shrink the tumors but merely delayed tumor growth (**Figure 4**). In a second colorectal model (CXF2102) and a breast model (HBCx-13B), FS102 also exhibited strong tumor control. This effect was superior to that of both trastuzumab and the combination of trastuzumab and pertuzumab. To demonstrate that the antitumor effect is mediated by specific HER2 targeting, a gastric and a breast PDX without HER2 amplification were subjected to the same treatment. No significant antitumor effect was observed from the treatments of any HER2 agents.

**Figure 5:**
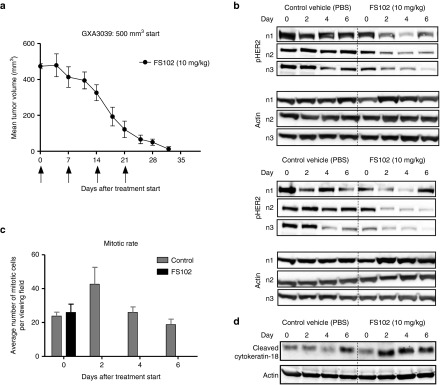
***Ex vivo* analyses of xenograft tumor tissues confirms the reduction of HER2 and the induction of apoptosis by FS102**. (**a**) FS102 treatment leads to complete tumor regression. Treatment administration schedule is shown by arrows. (**b**) Western blot of HER2 and pHER2 using tumor lysates collected from the mice on days 0, 2, 4, and 6. *n*1, *n*2, and *n*3 stand for three randomly selected mice within each treatment group. (**c**) Effect of FS102 on cell mitosis visualized by H&E staining of the tumor sections. Each column represents the average number of mitotic cells from 10 fields of view fields (error bar: standard deviation). (**d**) Western blot of cleaved cytokeratin-18 as an apoptosis marker. Each sample was a mixture of the tumor lysates collected from the three randomly selected mice within each treatment group. PBS, phosphate-buffered saline.

In a separate study, tumors from the GXA3039 gastric model were subjected to *ex vivo* analyses of HER2 receptor level and cell mitogenesis and apoptosis. Treatments were started with relatively larger tumors (500 mm^3^ or greater, as compared to 100–200 mm^3^ in a typical *in vivo* study) so that sufficient tumor tissue could be collected. FS102 maintained its unique activity to induce complete tumor regression even in these larger tumors (**Figure 5a**). Western blot analysis of the tumor lysates obtained from some of the mice 2, 4, or 6 days after the first dose showed that FS102 led to significant downregulation of both HER2 and pHER2 (**Figure 5b**). Consistent with the observations *in vitro*, FS102 increased the level of cleaved cytokeratin 18 in the treated tumors, indicating significant cell death in these samples (**Figure 5d**). This was accompanied by almost complete suppression of mitosis (**Figure 5c**). In all studies, the effect of FS102 was already apparent 48 hours after first dosing. Marked increase in cleaved cytokeratin 18 levels was also observed in the control tumor lysate. However, this increase was likely the result of background necrosis commonly observed in large tumors.

### Pharmacokinetics

The binding to neonatal Fc receptor (FcRn) contributes to the greater half-life of IgG1 due to recycling of the IgG1-FcRn complex to the cell surface. As mentioned earlier, the binding to FcRn was not affected by the mutations induced in the CH3 domain of FS102. Thus, the FcRn-mediated half-life protection may also apply to FS102. The half-lives of trastuzumab, FS102, and WT Fcab were determined in Balb/c mice and were found to be 428, 60, and 40 hours, respectively. The half-life of trastuzumab obtained was consistent with the reported range of 264–936 hours.^14^ The molecular weight of Fcabs (approximately 50 kDa) is in the range of the molecular weight cut off for glomerular filtration (30–60 kDa),^15^ therefore the fact that the half-lives of FS102 and the WT Fcab are shorter than that of trastuzumab is consistent with the difference in molecular weight of an Fcab and a mAb. However, the results confirm that the sequence changes in the CH3 domain of FS102 compared with the equivalent wild type Fc fragment do not adversely affect pharmacokinetics.

A dose-dependency study was conducted using a colorectal tumor model CXF 2102 to compare the potency of FS102 and trastuzumab. When both agents are dosed at 10 mg/kg, the molarity of the Fcab is approximately three times higher than that of trastuzumab. FS102 and trastuzumab showed T/C values of 9.0 and 89.8%, respectively. The T/C was 20.2% when the dosage of FS102 was reduced to 3 mg/kg to approximate the molarity of trastuzumab at the time of dosing. This however, does not take into account the somewhat faster clearance of FS102 (mouse *t*_½_ = 60 hours) compared with trastuzumab (mouse *t*_½_=428 hours). Thus, at once weekly equimolar dosing, the exposure (AUC) of FS102 is bound to be significantly lower than that of trastuzumab. Taking together, these results demonstrate that the superior efficacy of FS102 is not due to increased exposure.

### FS102 causes regression in tumors that are refractory to the combination of trastuzumab and pertuzumab

**Figure 6:**
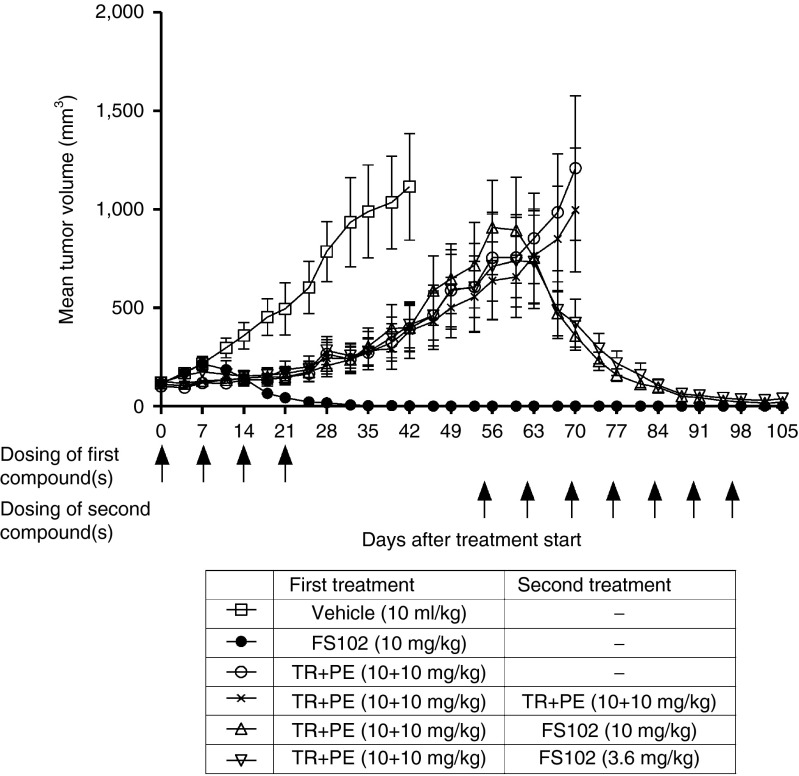
**FS102 causes complete tumor regression in patient-derived xenograft model that has progressed after the trastuzumab and pertuzumab combination treatment (TR+PE)**. Animals were subjected to four cycles of treatments with vehicle, FS102, or TR+PE. After 31-day recovery, they were subjected to seven additional cycles of treatments as indicated. Treatment administration schedule is shown by arrows.

The different biological activities of FS102 and the combination of trastuzumab and pertuzumab suggest that FS102 may be applied following the combination treatment to address intrinsic or acquired resistance. This was tested using the GXF281 model. In this study, mice underwent initial weekly treatments with vehicle control, FS102, or the combination of trastuzumab and pertuzumab for 4 weeks. As previously observed, FS102 induced complete tumor regression, while the combination treatment merely slowed the tumor growth compared to control. After a resting period, and when the tumors had reached an average size of approximately 600 mm^3^, all the animals in the trastuzumab and pertuzumab combination therapy group were randomized and either (i) continued on the combination therapy, or (ii) switched to FS102 treatment at either 10 or 3 mg/kg, or (iii) received no further treatment. Remarkably, the tumors which had progressed on the standard of care combination showed a complete response to FS102 at both dosages, whereas as expected they remained refractory to the combination (**Figure 6**).

### HER2 gene amplification status appears predictive of the antitumor effects of FS102

**Figure 7:**
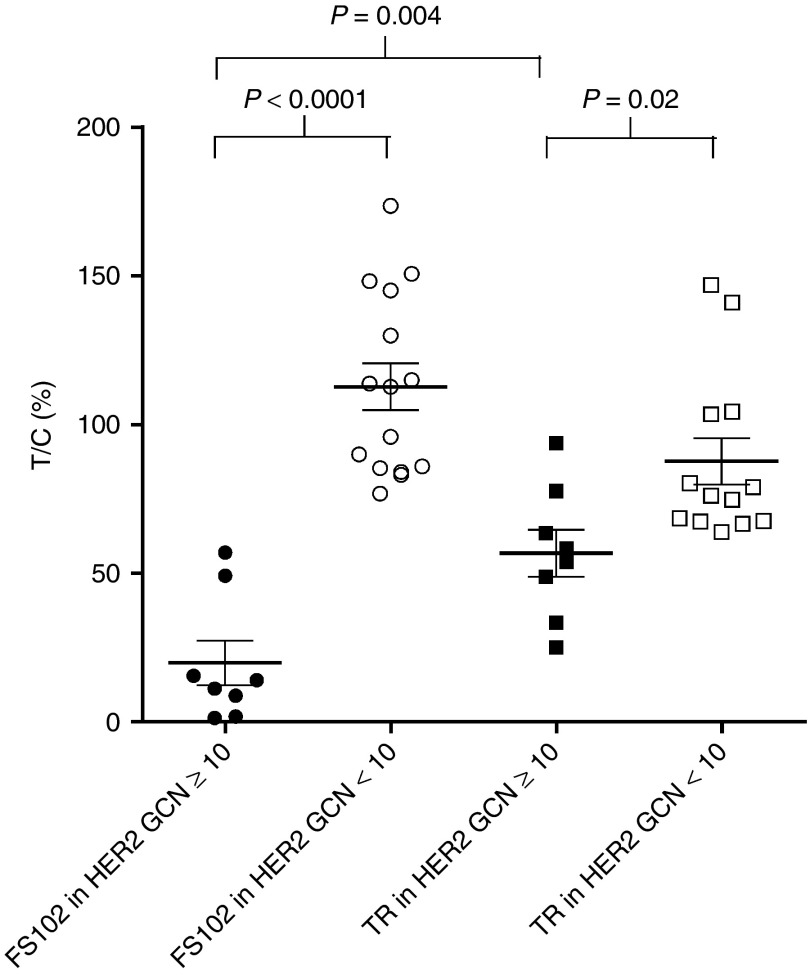
**FS102 shows potent activity in patient-derived xenograft (PDX) tumors with HER2 GCN ≥ 10**. Scatter plot showing T/C values of the PDX models treated with FS102 or trastuzumab (TR). *P* values were determined using unpaired Student's *T*-test. GCN, GCN, gene copy number; T/C, treatment/control.

Previous studies have demonstrated that the efficacy of trastuzumab is dependent on the HER2 amplification status of the patient.^16^ The effects of FS102 treatment in a total of 23 PDX tumors are summarized in **Table 1** and **Figure 7**. The PDX tumors displayed a wide range of HER2 gene copy number (GCN) per cell as measured by qPCR. A strong correlation is observed between the efficacy of FS102 and the HER2 GCN status of the tumors (**Figure 7**). In tumors with GCN ≥ 10 (8 out of 23 models), FS102 was not only active but showed efficacy that was consistently superior to that of trastuzumab. FS102 produced unusually potent antitumor effects in the majority of these tumors, as demonstrated by complete tumor regression in several models. In contrast, complete regression was never observed following trastuzumab treatment and only rarely with the trastuzumab and pertuzumab combination. In the xenograft tumors with HER2 GCN < 10, FS102 did not show significant antitumor effect. Interestingly, tumors in this group also responded poorly to trastuzumab.

## Discussion

FS102 belongs to a new class of biologics generated by introducing antigen-binding sites in the CH3 domains of the immunoglobulin constant domain.^11^ FS102 and the WT Fcab showed similar biophysical properties, suggesting no major structural deviation between the two molecules. The structure of the WT Fcab has been solved (data not shown) and is similar (root mean squared deviation (RMSD) = 0.3) to that of the reported structure of the IgG1 constant domain (1H3X). Based on this structure, it is inferred that the binding sites of FS102 form a relatively compact antibody fragment with two binding sites situated in close proximity. In contrast, the Fab arms of a typical mAb are separated by a flexible hinge region. By virtue of these structural differences, the two classes of molecules may target the same antigen differently, such as via different antigen epitopes or resulting in different configurations of the antigen:antibody complex. FS102 binds HER2 with high affinity comparable to that of trastuzumab and pertuzumab. However, it does not compete with either mAb in binding to the receptor.

Trastuzumab binds to HER2 on domain IV, an interaction that may provide a steric barrier to direct interaction of the transmembrane regions to avoid kinase activation.^17^ It facilitates endocytosis of HER2.^18^ Consequently, trastuzumab indirectly decreases the population of HER2 heterodimers by sequestering the monomer, even though it does not directly target the dimerization interface. The epitope recognized by trastuzumab is in close proximity to the proteolytic cleavage site on HER2 thus preventing shedding and the formation of constitutively active truncated receptors.^17,19,20^

Pertuzumab targets the dimerization of HER2, thereby inhibiting the formation of HER2 homodimers and HER2-containing heterodimers.^21,22^

Although the structure of the FS102:HER2 complex is not known, our data clearly suggest major differences in epitopes and the effects of formation of this complex compared with those of trastuzumab and pertuzumab. FS102 induces profound HER2 internalization and degradation, whereas trastuzumab internalizes but does not downregulate cell surface HER2.^18^ HER2 removal should inhibit the signaling of not only HER2 homodimers but also HER2-containing heterodimers because HER2 acts as the central node of the ERBB signaling pathways.^4^

Internalization and degradation of oncogenic receptors is an important mode of action for other antibody therapeutics (*e.g.*, against EGFR, c-MET, HER2, IGF1R).^23,24,25,26^ By removing the receptor from the cell surface, a therapy is less susceptible to certain resistance mechanisms such as compensatory ligand upregulation or ligand-independent activating mutations of the receptors.^27^ However, it has been suggested that HER2 homodimers and heterodimers are naturally resistant to internalization due to their exclusion from clathrin-coated pits, thereby avoiding the ubiquitin-c-Cbl-mediated lysosomal pathway for degradation.^28,29^ The current data showed that trastuzumab effectively internalized HER2, yet the internalization did not lead to equally effective removal of the receptor, consistent with a previous observation where trastuzumab-induced internalized HER2 is efficiently recycled.^18^ Recent data suggest that receptor internalization and degradation may be best achieved by a cocktail of antibodies or bispecific constructs that recognize different epitopes on the receptor and cause the formation of large antigen–antibody lattices.^30,31^ It is speculated that such complexes are prone to collapse into the cytoplasm and subsequent trafficking and degradation in the lysosome. Indeed, an EGFR-specific antibody cocktail has been demonstrated to be highly efficient in reducing EGFR signaling in cells that are resistant to ligand-blocking antibodies such as cetuximab and panitumumab.^27^

The two HER2 binding sites of FS102 are spatially close to each, as compared with those of a typical mAb (30–40 Å versus 120–170 Å).^32^ It is conceivable that the close proximity of the two sites in the relatively inflexible Fcab structure may favor a more ordered and tightly packed Fcab-antigen lattice and result in accelerated aggregation and internalization of the antigen/Fcab complexes. Alternatively, FS102 may act by inducing HER2 to adapt a conformation that is more susceptible to the degradation machinery. Such mechanism of action has been described previously where the antibody L26 interacted with HER2 to increase the recruitment of cbl.^33^ Further structural studies such as x-ray crystallography may be necessary to shed light on the internalization mechanism of the FS102:HER2 complex.

FS102 induces pronounced apoptosis in tumor cells expressing high level of HER2. Neither trastuzumab nor the combination of trastuzumab and pertuzumab show such an effect. Although the molecular events resulting from FS102 treatment have not been fully elucidated, apoptosis appears to be a key mechanism-of-action for the antitumor activity of FS102, which is consistent with the oncogene addiction paradigm. Overexpression of an oncogene growth receptor such as HER2 affords tumor cells with a growth advantage over the normal cells. Paradoxically, this advantage may also become the Achilles' heel of the tumor cell. Uncontrolled growth upsets cell homeostasis and exerts stress on the machinery that maintains normal physiological processes. Cancer cells cope with this pressure by activating prosurvival and antiapoptotic signals.^34^ This, together with other mutations and epigenetic abnormalities, renders the intracellular circuitry of the cancer cell in a bizarre state in which cancer cells become overtly dependent on the oncogene for survival. Profound inactivation and/or elimination of such an oncogene thus leads to “oncogene shock” and cell death due to differential attenuation of prosurvival and antiapoptotic signals.^35^ HER2 depletion as a way to induce oncogene shock has previously been demonstrated by using siRNA.^36^ In our studies, the oncogene shock may be more apparent in cells treated with FS102 than those by trastuzumab and pertuzumab due to the fast and profound HER2 elimination through internalization and degradation.

A pattern emerges when the biological activity of the Fcab is compared to the HER2 gene amplification status. By one measure the superior activity of FS102 over trastuzumab is observed when the HER2 gene copy number exceeds 10. Furthermore, our study shows that FS102 is capable of completely overcoming resistance to the combination therapy. These findings are consistent with our current understanding of the mechanism-of-action of the Fcab. Tumors with greater degrees of oncogene addiction are more likely to exhibit higher levels of HER2 gene amplification.^37^ The greater internalization and degradation induced by FS102 leads to receptor depletion and profound “oncogene shock” in oncogene-addicted tumors. Thus, in carefully selected tumors, FS102 may act as a cytotoxic rather than as a classical biologics therapeutic with growth inhibitory activity.

The development of HER2-specific agents represents one of the greatest achievements in targeted cancer therapy. Trastuzumab and pertuzumab have significantly improved the outlook for HER2-positive breast and gastric cancer patients.^38,39^ Recently, the trastuzumab-based antibody drug conjugate T-DM1 was approved in metastatic breast cancer, representing another significant milestone in targeting HER2. However, a number of resistance mechanisms to these standard-of-care agents have emerged. For example, trastuzumab is thought to derive some of its activity via ADCC, therefore patients with a weaker immune system may not respond well to trastuzumab-based treatment regimens.^40^ As T-DM1 becomes more broadly used, the incidence of resistance to T-DM1 may increase, in part because tumors that are insensitive to taxane-based chemotherapeutics are likely also to be resistant to T-DM1.^41^ Thus, a significant unmet medical need remains. FS102 is a novel HER2-specific biologic that induces significant tumor cell apoptosis, and its biological activity may be predicted by a clinically relevant biomarker.

The Fcab is remarkably stable and maintains many drug-like features of a mAb. In PDX mouse tumor models, FS102 significantly reduces the growth of breast, gastric and colorectal cancers which are positive for the HER2 gene copy number biomarker, in many cases leading to complete tumor regression. Thus, FS102 holds significant promise as a biomarker-driven clinical candidate in a subset of solid tumor patients with high HER2 gene copy numbers.

## Materials and Methods

***Library design and construction.*** The Fcab library was constructed using the modified pYD1 (Life Technologies, Carlsbad, CA) yeast display vector pYD1dXFcdel as described previously.^11^ Briefly, residues 358–362 of the AB loop and 413–419 of the EF loop (Kabat EU numbering)^13^ in the CH3 domain were randomized by PCR using NNB-oligonucleotides.^11^ Growth of the libraries, induction of display, and determination of library size were performed according to standard protocols (Life Technologies). Libraries and clones were monitored for display using anti-Xpress antibody (Life Technologies) and for the quality of displayed protein using anti-CH2-FITC (AbD Serotec, Oxford, UK). Induction was carried out at 37 °C during different stages of construction (**Supplementary Figure S3a**).^42^ Functional clones were gated based on binding to anti-Xpress and anti-CH2 antibodies. These clones were isolated and used as a template for an additional iteration of library construction in order to reach desired diversity (**Supplementary Figure S3b**). The final naive library contains >10^8^ independent clones.

***HER2 selections and screening.*** Selection and screening of Fcab libraries to HER2-ECD (Bender MedSystems, Vienna, Austria) were performed as described previously.^11,43^ Positive binders were detected with streptavidin-R-phycoerythrin (Sigma, St Louis, MO). The selection was repeated three times against 500 nmol/l biotinylated HER2 ECD and initial hits were subjected to additional affinity maturation by rerandomizing the AB or EF loop. All 20 amino acids were represented equally for each position during the randomization. The resulting library was subjected to another three rounds of selection against 50 nmol/l biotinylated HER2 ECD and positive clones were expressed as soluble Fcabs. FS102 was selected based on its overall superior attributes in affinity and specificity to HER2 ECD.

***Cell lines, animals, and reagents.*** Human breast cancer cell lines BT-474, SK-BR-3, HCC1954, and MCF-7 were obtained from the American Type Culture Collection (ATCC, Manassas, VA). Antitumor effects were assessed in vivo using PDX. Models of gastric tumors (GXA3039, GXF281, GXA3054, GXA3038, GXA3067, and GXA3005) and colorectal tumors (CXF1991, CXF2102, CXF647, CXF1103, CXF975, CXF1729, CXF260, and CXF1034) breast tumors (MAXF583 and MAXF508), lung tumors (LXFA983 and LXFE690), ovarian tumor (OVXF1023), and pancreatic tumors (PAXF736 and PAXF2005)) were provided by Oncotest (Freiburg, Germany). The breast tumor model HBCx-13B was provided by Xentech (Evry, France) and the gastric tumor model GA0060 was provided by Crown Bioscience (Taicang, China).

***BIAcore analyses.*** Data were acquired using a BIAcore 3000. All dilution mixtures were prepared in HBS-P buffer (GE Healthcare, Little Chalfont, UK). Antigens were immobilized on CM5 chips using the standard amine coupling technique. For *K*_D_ determination, the antigen human HER2 ECD-Fc recombinant (R&D Systems, Minneapolis, MN) was immobilized on a CM5 chip to 200 response units (RU). The analyte FS102 (50 µg/ml) was injected at a rate of 20 µl/minute. For specificity assessment, the human EGFR, HER3, or HER4 ECD-Fc antigens (R&D systems, Minneapolis, MN) were immobilized on a CM5 chip to 6,000 RU. The analyte FS102 (50 µg/ml) was injected at 20 µl/minute. For competition binding assessment, the human HER2 ECD-Fc antigen was immobilized on a CM5 chip to 1,000 RU. The two analytes were injected at a flow rate of 20 µl/minute sequentially: the first contained one anti-HER2 agent (10 µg/ml) and was injected to saturate the immobilized antigen while the second is a mixture of the first agent and a competing agent (both at 10 µg/ml).

***Flow cytometry.*** Cells were seeded at 2 × 10^6^ cells/ml in 96-well plates in cold buffer (1× Dulbecco's phosphate-buffered saline, calcium and magnesium, Life Technologies) containing 2% fetal bovine serum (FBS, Life Technologies) and incubated with 0.1–2,000 nmol/l primary antibodies or Fcabs for 1 hour at 4 °C with agitation. Binding was detected by an anti-Human IgG-Phycoerythrin secondary antibody (P8047, Sigma) according to the standard protocol.

***Internalization of Fcabs and antibodies.*** BT474 cells were seeded in a 96-well plate at 3 × 10^4^ cells/well. 5 µg/ml of Alexa-488 conjugated antibodies or Fcabs (Lightning-Link Rapid DyLight 488, Innova Bioscience, Cambridge, UK) were added to the cells. After 45 minutes of incubation on ice, cells were washed before subjected to a time course study: Cells were incubated at either 4 or 37 °C, and harvested at given time points. To stop the incubation, cells were fixed in 3% formaldehyde (Sigma, St Louis, MO). Fixed cells were stained by Hoechst (H3570, Life Technologies), and imaged at 20× magnification using ImageXpress MicroXL (Molecular Devices, Sunnyvale, CA).

***HER2 imaging.*** SK-BR-3 cells were seeded in a 96-well plate at 1 × 10^4^ cells/well in McCoy's 5A medium containing 10% FBS and incubated at 37 °C overnight. Each test article was added the next day to the cells at a final concentration of 200 nmol/l. The cells were incubated at 37 °C for an additional 24, 48, or 72 hours, then washed with phosphate-buffered saline (PBS) and fixed with 4% formaldehyde in PBS for 20 minutes. To detect membrane HER2, cells were subjected to HER2 staining using an anti-HER2 primary antibody (MGR2, Enzo Life Sciences, Farmingdale, NY) and an anti-mouse secondary antibody (anti-mouse Alexa-647, Life Technologies). Cell number was determined using Hoechst nuclear staining. To detect total HER2 (both membrane and cytosolic proteins), cells were permeabilized using 0.1% TritonX100 in PBS and 0.1 mg/ml glucose and subjected to HER2 staining. Imaging and data analysis were conducted using an ImageXpress MicroXL using 40× magnification. Quantification of percentage of cell displaying HER2 was determined using:


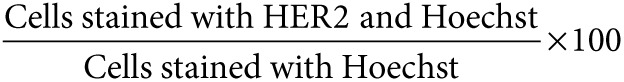


***Western blot.*** SK-BR-3 cells were seeded in a six-well plate at 3.3 × 10^5^ cells/ml in McCoy's 5A medium containing 0.1% FBS. Each test article was added to a final concentration of 200 nmol/l and incubated at 37 °C for 24, 48, or 72 hours. Cells were lysed and subjected to western blot analysis. The following primary antibodies were used: anti-HER2 (#2248, Cell Signaling Technology, Danvers, MA), anti-pHER2 (#2243, Cell Signaling Technology), and anti-β-actin (A1978, Sigma). Anti-mouse and anti-rabbit secondary antibodies were from Jackson ImmunoResearch (West Grove, PA).

***Proliferation.*** Unless specified otherwise, tumor cells were cultured at 37 °C in growth media containing 10% FBS. SK-BR-3 and HCC1954 cells were seeded overnight in 96-well plates at 7.5 × 10^3^ and 1 × 10^3^ cells/well, respectively. Test articles were added and treatment lasted for 5 days at 37 °C_._ At the end of study, cells in each well were counted.

***Apoptosis assays.*** SK-BR-3 cells were treated for 5 days as described above. At the end of the treatment, cells were stained at room temperature (RT) for 1 hour with a mixture of 1 µg/ml Hoechst, 2.5 µg/ml propidium iodide (PI, P4864, Sigma), and 1% Alexa-647 Annexin V (Life technologies). Stained cells were imaged at 40× magnification. The staining of Annexin V and PI were used to quantify late apoptotic (Annexin V+/PI+) and necrotic cells (Annexin V-/PI+). Caspase 3/7 activity was measured using the Promega (Fitchburg, WI) Caspase-Glo 3/7 assay following the manufacturer's instructions.

***DNA copy number analysis.*** Quantitative PCR analysis (qPCR) on DNA isolated from PDX models was performed using Taqman Genotyping Master Mix (#4371355, Life Technologies), HER2 Taqman primers (Gene Assay ID: Hs00817646 Cat No:4400291, Life Technologies) and housekeeping RNAseP Taqman primers (#4403326, Life Technologies). After acquisition of raw cycle threshold (*C*_T_) data, a manual *C*_T_ of 0.2 and an auto-baseline were applied to yield the final results.

All data were normalized to the housekeeping gene. The results of the quantitative PCR were expressed in arbitrary units (AU, related to the copy number per cell). The difference between the *C*_T_ value of a housekeeping gene and the HER2 gene was calculated as follows:

Target gene expression = 2 (*C*_T_ housekeeping gene – *C*_T_ HER2 gene)

Results were analyzed using Copy Caller Software (Life Technologies). A DNA reference sample (#11691112001, Roche Life Science, Indianapolis, IN) was included as a standard based on which results are normalized (normal value for HER2 gene in the reference genomic DNA = 2 AU).

***PDX models.*** PDX tumors were implanted subcutaneously (s.c.) in Balb/c nude mice (Crown Bioscience), NMRI nu/nu mice (Oncotest), or athymic nude-Foxn1^nu^ mice (XenTech). Test articles were delivered intravenously (i.v.). Animals were cared for following local, national, and international guidelines. Tumor inhibition was quantified as *T*/*C* value, defined as the ratio (in percentage) of the mean tumor volume of a test article versus a control on any given day.

***Tumor tissue analyses.*** GXA3039 tumors were grown in nude mice to the size of ~500 mm^2^. Mice were randomized and subjected to i.v. treatment of test articles on day 0 and day 3. Three mice in each treatment group were randomly selected and sacrificed on day 0 (before treatment), day 2, day 4, and day 6. Tumors were excised and prepared into lysates or tissue sections (4 µmol/l thickness). Lysates were analyzed by western blot. Anti-HER2 (#2242, Cell Signaling Technology), anti-pHER2 (#2243, Cell Signaling Technology), anti-M30 (12140322001, Roche Life Science, Indianapolis, IN), and anti-actin (A2228, Sigma) were used as primary antibodies. Anti-rabbit-horseradish peroxidase and anti-mouse-horseradish peroxidase secondary antibodies were from Jackson ImmunoResearch (West Grove, PA). H&E staining was performed on tumor sections according to standard protocols. Mitotic rate was scored by counting the number of cells undergoing mitosis in ten randomly selected fields of the tumor sections under 40× magnification.

***Pharmacokinetics.*** A single intravenous injection of 10 mg/kg purified WT Fcab, FS102, or trastuzumab was administered into the tail vein of Balb/c mice. Blood samples were taken from the retro-orbital plexus using haematocrit capillary tubes before injection and at multiple time points between 0.25–144 hours after injection. The serum concentration of WT Fcab, FS102, or trastuzumab was measured by the detection of human IgG1 Fc using an ELISA protocol. A F(ab')_2_ fragment directed against human Fc (Sigma) was coated at 1 µg/ml overnight. Serial dilutions of sera were added and incubated for 1 hour at room temperature. Bound human IgG1 Fc were detected with Protein A coupled to horseradish peroxidase for 30 minutes. The standard curve was established by spiking WT Fcab, FS102, or trastuzumab into the mouse serum in a dilution series from 2,000 to 0.98 ng/ml in 2% bovine serum albumin. The serum concentration of WT Fcab, FS102, or trastuzumab was interpolated from the standard curve.

***Quantification of antibody binding capacity.*** Quantum Simply Cellular anti-human IgG microbead kit (BLI816, Bangs Laboratories, Fishers, IN) was adopted to quantify the antibody binding capacity of the cells (SK-BR-3, HCC1954, and CHO-K1). The experiments were conducted according to the protocols provided by the manufacturer of the kit. Fluorochrome-labeled antibodies were incubated with the cells at saturating conditions (1 µmol/l) for 1 hour at 4 °C. Bound antibodies were subjected to flow cytometry analyses. Raw data were processed by interpolation of a standard curve to yield antibody binding capacity values.

**SUPPLEMENTARY MATERIAL** **Figure S1.** Biophysical properties of FS102 as evaluated by size exclusion chromatography (SEC) and differential scanning calorimetry (DSC) thermograms. **Figure S2.** Binding specificity and quantification of antibody binding sites of FS102. **Figure S3.** Flow cytometry profiles of an Fcab yeast surface display library. **Table S1.** Binding of FS102 to Fc receptors **Table S2.** Binding of HER2 targeting biologics to HER2 ECD

## Figures and Tables

**Table 1:**
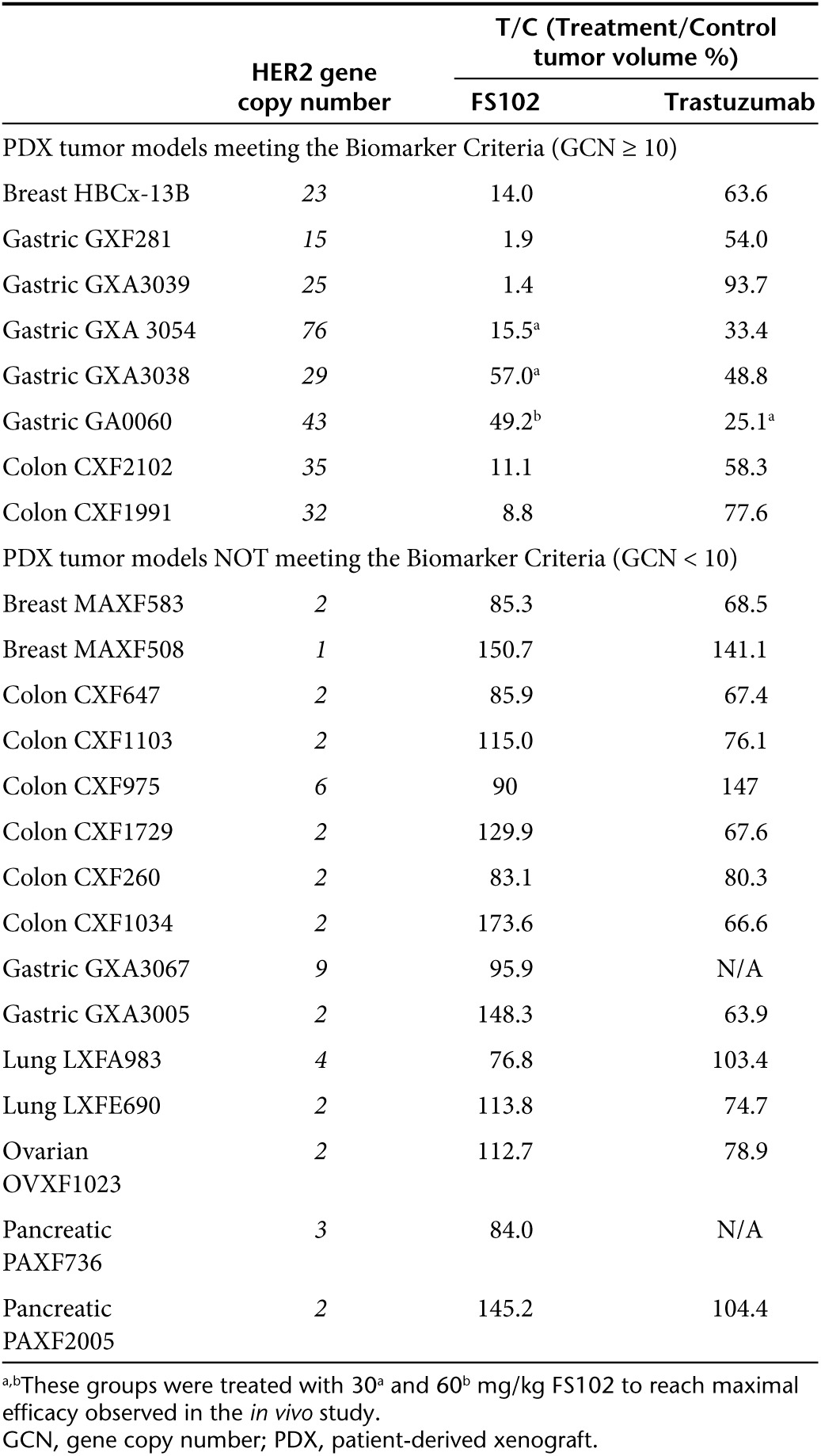
Summary of the efficacy as defined by the T/C values of FS102 and trastuzumab treatments and HER2 GCN of the 23 PDX models tested

## References

[bib1] Hynes, NE and Lane, HA (2005). ERBB receptors and cancer: the complexity of targeted inhibitors. Nat Rev Cancer 5: 341–354. 15864276 10.1038/nrc1609

[bib2] Klapper, LN, Glathe, S, Vaisman, N, Hynes, NE, Andrews, GC, Sela, M et al. (1999). The ErbB-2/HER2 oncoprotein of human carcinomas may function solely as a shared coreceptor for multiple stroma-derived growth factors. Proc Natl Acad Sci U S A 96: 4995–5000. 10220407 10.1073/pnas.96.9.4995 PMC21805

[bib3] Garrett, TP, McKern, NM, Lou, M, Elleman, TC, Adams, TE, Lovrecz, GO et al. (2003). The crystal structure of a truncated ErbB2 ectodomain reveals an active conformation, poised to interact with other ErbB receptors. Mol Cell 11: 495–505. 12620236 10.1016/s1097-2765(03)00048-0

[bib4] Graus-Porta, D, Beerli, RR, Daly, JM and Hynes, NE (1997). ErbB-2, the preferred heterodimerization partner of all ErbB receptors, is a mediator of lateral signaling. EMBO J 16: 1647–1655. 9130710 10.1093/emboj/16.7.1647 PMC1169769

[bib5] Baulida, J, Kraus, MH, Alimandi, M, Di Fiore, PP and Carpenter, G (1996). All ErbB receptors other than the epidermal growth factor receptor are endocytosis impaired. J Biol Chem 271: 5251–5257. 8617810 10.1074/jbc.271.9.5251

[bib6] Ross, JS and Fletcher, JA (1998). The HER-2/neu Oncogene in Breast Cancer: Prognostic Factor, Predictive Factor, and Target for Therapy. Oncologist 3: 237–252. 10388110

[bib7] Okines, AF and Cunningham, D (2010). Trastuzumab in gastric cancer. Eur J Cancer 46: 1949–1959. 20542421 10.1016/j.ejca.2010.05.003

[bib8] Slamon, DJ, Clark, GM, Wong, SG, Levin, WJ, Ullrich, A and McGuire, WL (1987). Human breast cancer: correlation of relapse and survival with amplification of the HER-2/neu oncogene. Science 235: 177–182. 3798106 10.1126/science.3798106

[bib9] Gravalos, C and Jimeno, A (2008). HER2 in gastric cancer: a new prognostic factor and a novel therapeutic target. Ann Oncol 19: 1523–1529. 18441328 10.1093/annonc/mdn169

[bib10] Verma, S, Miles, D, Gianni, L, Krop, IE, Welslau, M, Baselga, J et al.; EMILIA Study Group. (2012). Trastuzumab emtansine for HER2-positive advanced breast cancer. N Engl J Med 367: 1783–1791. 23020162 10.1056/NEJMoa1209124 PMC5125250

[bib11] Wozniak-Knopp, G, Bartl, S, Bauer, A, Mostageer, M, Woisetschläger, M, Antes, B et al. (2010). Introducing antigen-binding sites in structural loops of immunoglobulin constant domains: Fc fragments with engineered HER2/neu-binding sites and antibody properties. Protein Eng Des Sel 23: 289–297. 20150180 10.1093/protein/gzq005

[bib12] Hasenhindl, C, Traxlmayr, MW, Wozniak-Knopp, G, Jones, PC, Stadlmayr, G, Rüker, F et al. (2013). Stability assessment on a library scale: a rapid method for the evaluation of the commutability and insertion of residues in C-terminal loops of the CH3 domains of IgG1-Fc. Protein Eng Des Sel 26: 675–682. 24006374 10.1093/protein/gzt041 PMC3785252

[bib13] US Department of Health and Human Services. Sequences of proteins of immunological interest, 1991. Report 91-3242. Bethesda, MD: US.

[bib14] European Medicines Agency. Herceptin: EPAR - Scientific Discussion. 2005. London, UK.

[bib15] Deen, WM, Lazzara, MJ and Myers, BD (2001). Structural determinants of glomerular permeability. Am J Physiol Renal Physiol 281: F579–F596. 11553505 10.1152/ajprenal.2001.281.4.F579

[bib16] Gomez-Martin, C, Plaza, JC, Pazo-Cid, R, Salud, A, Pons, F, Fonseca, P et al. (2013). Level of HER2 gene amplification predicts response and overall survival in HER2-positive advanced gastric cancer treated with trastuzumab. J Clin Oncol 31: 4445–4452. 24127447 10.1200/JCO.2013.48.9070

[bib17] Cho, HS, Mason, K, Ramyar, KX, Stanley, AM, Gabelli, SB, Denney, DW Jr et al. (2003). Structure of the extracellular region of HER2 alone and in complex with the Herceptin Fab. Nature 421: 756–760. 12610629 10.1038/nature01392

[bib18] Austin, CD, De Mazière, AM, Pisacane, PI, van Dijk, SM, Eigenbrot, C, Sliwkowski, MX et al. (2004). Endocytosis and sorting of ErbB2 and the site of action of cancer therapeutics trastuzumab and geldanamycin. Mol Biol Cell 15: 5268–5282. 15385631 10.1091/mbc.E04-07-0591 PMC532009

[bib19] Molina, MA, Codony-Servat, J, Albanell, J, Rojo, F, Arribas, J and Baselga, J (2001). Trastuzumab (herceptin), a humanized anti-Her2 receptor monoclonal antibody, inhibits basal and activated Her2 ectodomain cleavage in breast cancer cells. Cancer Res 61: 4744–4749. 11406546

[bib20] Yuan, CX, Lasut, AL, Wynn, R, Neff, NT, Hollis, GF, Ramaker, ML et al. (2003). Purification of Her-2 extracellular domain and identification of its cleavage site. Protein Expr Purif 29: 217–222. 12767812 10.1016/s1046-5928(03)00058-5

[bib21] Agus, DB, Akita, RW, Fox, WD, Lewis, GD, Higgins, B, Pisacane, PI et al. (2002). Targeting ligand-activated ErbB2 signaling inhibits breast and prostate tumor growth. Cancer Cell 2: 127–137. 12204533 10.1016/s1535-6108(02)00097-1

[bib22] Diermeier-Daucher, S, Hasmann, M and Brockhoff, G (2008). Flow cytometric FRET analysis of erbB receptor interaction on a cell-by-cell basis. Ann N Y Acad Sci 1130: 280–286. 18596360 10.1196/annals.1430.003

[bib23] Sunada, H, Magun, BE, Mendelsohn, J and MacLeod, CL (1986). Monoclonal antibody against epidermal growth factor receptor is internalized without stimulating receptor phosphorylation. Proc Natl Acad Sci USA 83: 3825–3829. 2424012 10.1073/pnas.83.11.3825 PMC323616

[bib24] Greenall, SA, Gherardi, E, Liu, Z, Donoghue, JF, Vitali, AA, Li, Q et al. (2012). Non-agonistic bivalent antibodies that promote c-MET degradation and inhibit tumor growth and others specific for tumor related c-MET. PLoS One 7: e34658. 22511956 10.1371/journal.pone.0034658 PMC3325269

[bib25] Ren, XR, Wei, J, Lei, G, Wang, J, Lu, J, Xia, W et al. (2012). Polyclonal HER2-specific antibodies induced by vaccination mediate receptor internalization and degradation in tumor cells. Breast Cancer Res 14: R89. 22676470 10.1186/bcr3204 PMC3446352

[bib26] Wang, Y, Hailey, J, Williams, D, Wang, Y, Lipari, P, Malkowski, M et al. (2005). Inhibition of insulin-like growth factor-I receptor (IGF-IR) signaling and tumor cell growth by a fully human neutralizing anti-IGF-IR antibody. Mol Cancer Ther 4: 1214–1221. 16093437 10.1158/1535-7163.MCT-05-0048

[bib27] Pedersen, MW, Jacobsen, HJ, Koefoed, K, Hey, A, Pyke, C, Haurum, JS et al. (2010). Sym004: a novel synergistic anti-epidermal growth factor receptor antibody mixture with superior anticancer efficacy. Cancer Res 70: 588–597. 20068188 10.1158/0008-5472.CAN-09-1417

[bib28] Hommelgaard, AM, Lerdrup, M and van Deurs, B (2004). Association with membrane protrusions makes ErbB2 an internalization-resistant receptor. Mol Biol Cell 15: 1557–1567. 14742716 10.1091/mbc.E03-08-0596 PMC379255

[bib29] Shen, F, Lin, Q, Childress, C and Yang, W (2008). Identification of the domain in ErbB2 that restricts ligand-induced degradation. Cell Signal 20: 779–786. 18255265 10.1016/j.cellsig.2007.12.021

[bib30] Ben-Kasus, T, Schechter, B, Lavi, S, Yarden, Y and Sela, M (2009). Persistent elimination of ErbB-2/HER2-overexpressing tumors using combinations of monoclonal antibodies: relevance of receptor endocytosis. Proc Natl Acad Sci USA 106: 3294–3299. 19218427 10.1073/pnas.0812059106 PMC2651295

[bib31] Boersma, YL, Chao, G, Steiner, D, Wittrup, KD and Plückthun, A (2011). Bispecific designed ankyrin repeat proteins (DARPins) targeting epidermal growth factor receptor inhibit A431 cell proliferation and receptor recycling. J Biol Chem 286: 41273–41285. 21979953 10.1074/jbc.M111.293266 PMC3308840

[bib32] Doores, KJ, Fulton, Z, Huber, M, Wilson, IA and Burton, DR (2010). Antibody 2G12 recognizes di-mannose equivalently in domain- and nondomain-exchanged forms but only binds the HIV-1 glycan shield if domain exchanged. J Virol 84: 10690–10699. 20702629 10.1128/JVI.01110-10 PMC2950587

[bib33] Klapper, LN, Waterman, H, Sela, M and Yarden, Y (2000). Tumor-inhibitory antibodies to HER-2/ErbB-2 may act by recruiting c-Cbl and enhancing ubiquitination of HER-2. Cancer Res 60: 3384–3388. 10910043

[bib34] Petry, IB, Fieber, E, Schmidt, M, Gehrmann, M, Gebhard, S, Hermes, M et al. (2010). ERBB2 induces an antiapoptotic expression pattern of Bcl-2 family members in node-negative breast cancer. Clin Cancer Res 16: 451–460. 20068093 10.1158/1078-0432.CCR-09-1617

[bib35] Weinstein, IB and Joe, A (2008). Oncogene addiction. Cancer Res 68: 3077–80; discussion 3080. 18451130 10.1158/0008-5472.CAN-07-3293

[bib36] Choudhury, A, Charo, J, Parapuram, SK, Hunt, RC, Hunt, DM, Seliger, B et al. (2004). Small interfering RNA (siRNA) inhibits the expression of the Her2/neu gene, upregulates HLA class I and induces apoptosis of Her2/neu positive tumor cell lines. Int J Cancer 108: 71–77. 14618618 10.1002/ijc.11497

[bib37] Shiu, KK, Wetterskog, D, Mackay, A, Natrajan, R, Lambros, M, Sims, D et al. (2014). Integrative molecular and functional profiling of ERBB2-amplified breast cancers identifies new genetic dependencies. Oncogene 33: 619–631. 23334330 10.1038/onc.2012.625

[bib38] Bang, YJ, Van Cutsem, E, Feyereislova, A, Chung, HC, Shen, L, Sawaki, A et al.; ToGA Trial Investigators. (2010). Trastuzumab in combination with chemotherapy versus chemotherapy alone for treatment of HER2-positive advanced gastric or gastro-oesophageal junction cancer (ToGA): a phase 3, open-label, randomised controlled trial. Lancet 376: 687–697. 20728210 10.1016/S0140-6736(10)61121-X

[bib39] Swain, SM, Kim, SB, Cortés, J, Ro, J, Semiglazov, V, Campone, M et al. (2013). Pertuzumab, trastuzumab, and docetaxel for HER2-positive metastatic breast cancer (CLEOPATRA study): overall survival results from a randomised, double-blind, placebo-controlled, phase 3 study. Lancet Oncol 14: 461–471. 23602601 10.1016/S1470-2045(13)70130-X PMC4076842

[bib40] Clynes, RA, Towers, TL, Presta, LG and Ravetch, JV (2000). Inhibitory Fc receptors modulate *in vivo* cytotoxicity against tumor targets. Nat Med 6: 443–446. 10742152 10.1038/74704

[bib41] Phillips, GD, Fields, CT, Li, G, Dowbenko, D, Schaefer, G, Miller, K et al. (2014). Dual targeting of HER2-positive cancer with trastuzumab emtansine and pertuzumab: critical role for neuregulin blockade in antitumor response to combination therapy. Clin Cancer Res 20: 456–468. 24097864 10.1158/1078-0432.CCR-13-0358

[bib42] Shusta, EV, Holler, PD, Kieke, MC, Kranz, DM and Wittrup, KD (2000). Directed evolution of a stable scaffold for T-cell receptor engineering. Nat Biotechnol 18: 754–759. 10888844 10.1038/77325

[bib43] Chao, G, Lau, WL, Hackel, BJ, Sazinsky, SL, Lippow, SM and Wittrup, KD (2006). Isolating and engineering human antibodies using yeast surface display. Nat Protoc 1: 755–768. 17406305 10.1038/nprot.2006.94

