# Supplementary material for: A HER2-specific Modified Fc Fragment (Fcab) Induces Antitumor Effects Through Degradation of HER2 and Apoptosis
Source: Mol Ther. 2015 Aug 25;23(11):1722–33. doi: 10.1038/mt.2015.127 (PMC4817942; doi:10.1038/mt.2015.127)
Supplement: Supplementary Figures and Tables [file mt2015127x1.pdf]

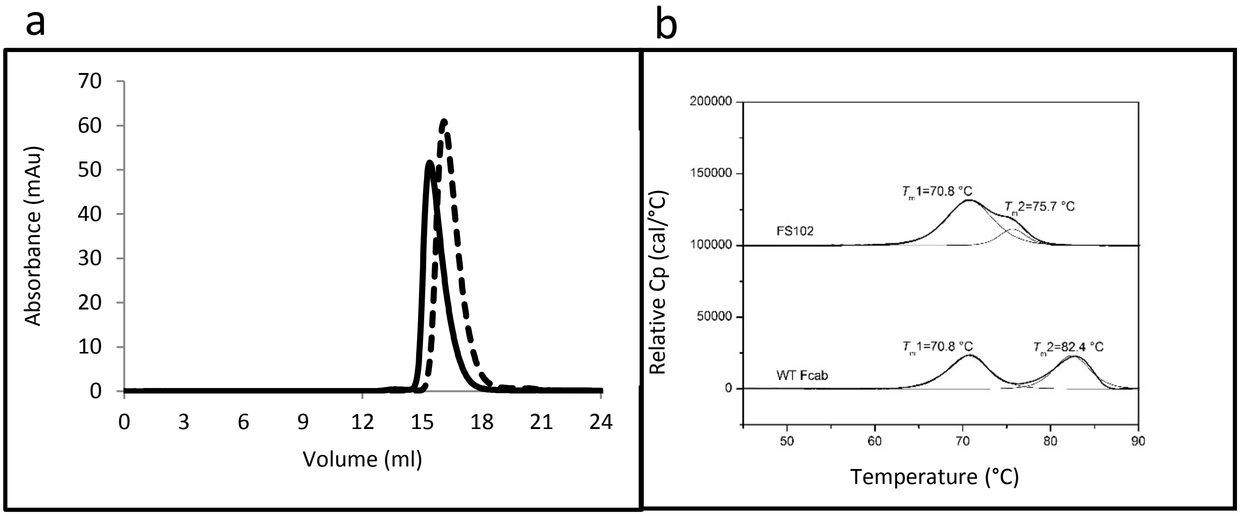

Supplementary Figure S1: Biophysical properties of FS102 as evaluated by size exclusion chromatography (SEC) and differential scanning calorimetry (DSC) thermograms. (A) Approximately 250 µg of FS102 (Dash line) and WT Fcab (solid line) were subjected to SEC using a Superdex 200 column (10 x 300 mm, GE Healthcare) at 0.4 ml/min in PBS. (B) Thermal transitions (T_m1 and T_m2) of FS102 and WT Fcab were monitored at a heating rate of 1 °C/min to analyse the thermal stability. The excess heat capacity (C_p) was shown as a function of temperature. The baseline was recorded using denatured unfolded FS102 and WT Fcab. After subtraction of the baseline, data were normalized for protein concentration and fitted according to a non-2-state thermal unfolding model using the software Origin 7 (MicroCal). T_m1 is due to the unfolding of the CH2 domain which has an identical sequence in FS102 and WT Fcab and therefore an identical T_m. T_m2 is due to the unfolding of the CH3 domain. The CH3 of FS102 unfolds at 6.7 °C lower than that of WT Fcab.

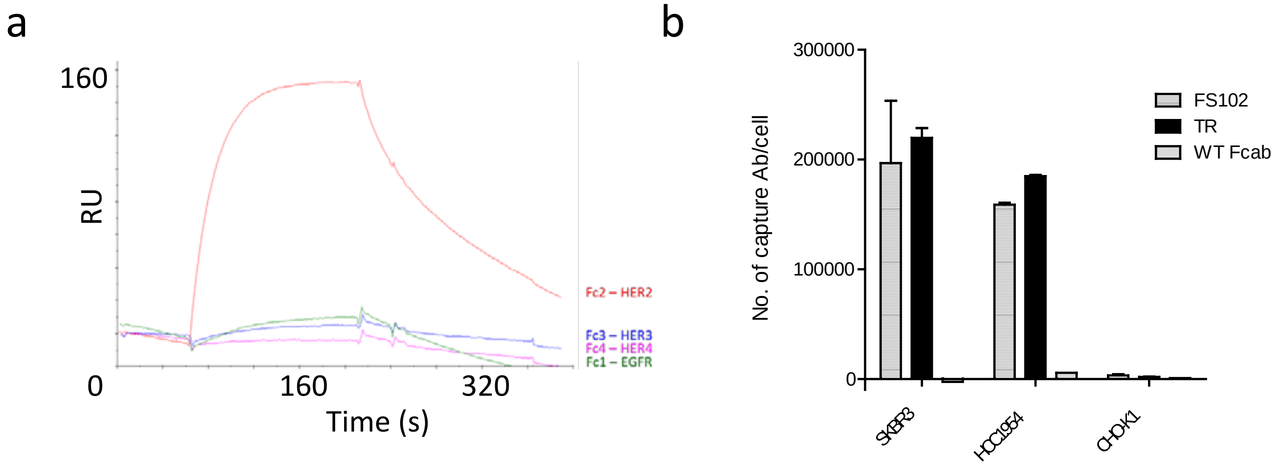

Supplementary Figure S2: Binding specificity and quantification of antibody binding sites of FS102. (a) Sensorgram shows the binding of FS102 to HER receptor family recombinant proteins by BIAcore. Flow cell 1 (Fc1): EGFR; Fc2: HER2; Fc3: HER3; Fc4: HER4. (b) Quantification of antibody binding sites (ABC) on the cell surface. Bead-based calibration of antibody binding sites on SK-BR-3, HCC1954 or CHO-K1 cells determined by flow cytometry. Each data point is the average of duplicated samples (error bar: standard deviation).

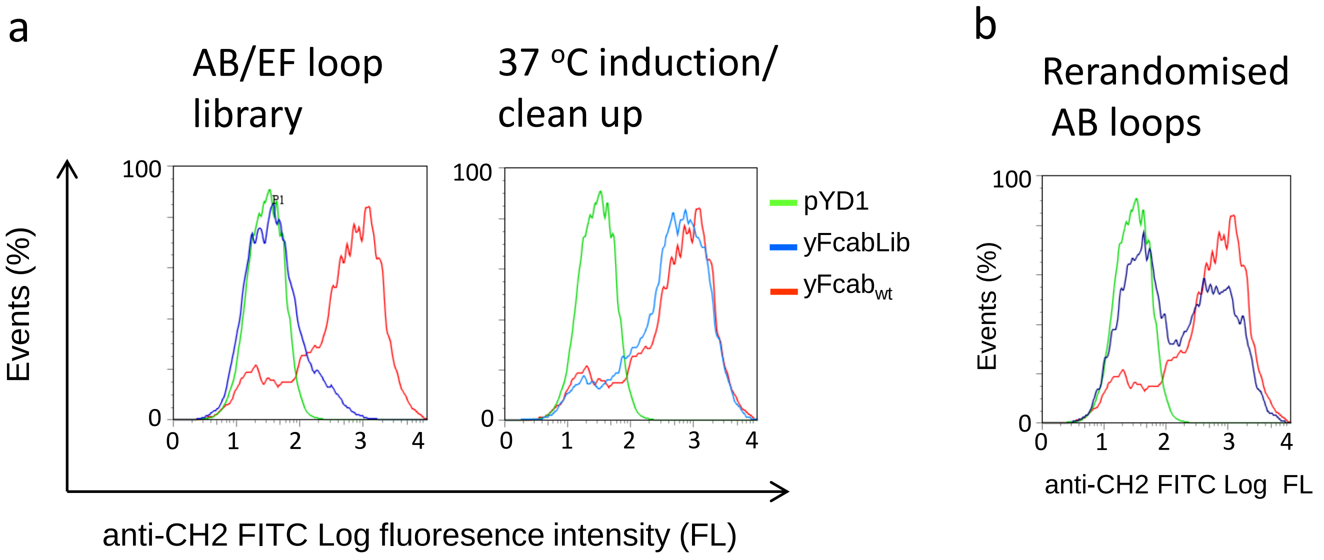

Supplementary Figure S3: (a) Flow cytometry profiles of an Fcab yeast surface display library before (left plot) and after (right plot) high temperature induction and anti-CH2 selection. (b) The effect of re-randomising the AB loop of a library selected after high temperature induction and staining with anti-CH2.

Affinity to Fc receptor (K_D, nM)	IgG_1	FS102	WT Fcab	Trastuzumab
**FcγRI (CD64)**	15	19 ± 2	27 ± 12	40 ± 14
**FcγRIIA (CD32A)**	250	472 ± 200	1000± 280	560± 330
**FcγRIIB (CD32B)**	5000	6400 ± 800	6200 ± 1400	5100 ± 4600
**FcγRIII (CD16A)**	660	560 ± 130	500 ± 250	410 ± 260
**FcRn***	20-60	45 ± 12	46 ± 12	33 ± 15

Supplementary Table S1: Binding of FS102, WT Fcab, trastuzumab and IgG to Fc receptors determined by Biacore analysis. *FcRn data collected at pH6

Kinetic data to HER2	k_{on} ($M^{-1}s^{-1}$)	k_{off} (s^{-1})	K_D (nM)
**FS102**	$5.9 \times 10^5 \pm 1.5 \times 10^5$	$4.7 \times 10^{-4} \pm 0.9 \times 10^{-4}$	0.8 ± 0.3
**Trastuzumab**	$4.9 \times 10^5 \pm 6.4 \times 10^4$	$1.7 \times 10^{-4} \pm 1.8 \times 10^{-5}$	0.4 ± 0.03
**Pertuzumab**	$9.8 \times 10^4 \pm 1.7 \times 10^4$	$6.3 \times 10^{-4} \pm 2.0 \times 10^{-4}$	6.3 ± 0.9

Supplementary Table S2: Binding of HER2 targeting biologics to HER2 ECD
